# Case report: acute facial swelling in a recreational technical diver

**DOI:** 10.14814/phy2.13240

**Published:** 2017-03-31

**Authors:** Peter Buzzacott, William K. Dolen, James Chimiak

**Affiliations:** ^1^ Divers Alert NetworkDurhamNorth Carolina; ^2^ School of Sports ScienceExercise and HealthUniversity of Western AustraliaCrawleyWestern AustraliaAustralia; ^3^ Medical College of Georgia at Augusta UniversityAugustaGeorgia

**Keywords:** Allergy, immunology, latex, rubber, scuba

## Abstract

A recreational scuba diver wore a second scuba regulator against his face during a scuba dive, attached by an elastic rubber cord necklace. After surfacing, the diver's left face became swollen. Through a process of elimination all other items of scuba equipment were excluded as potential causes. A dive with the same equipment minus the necklace confirmed the involvement of the necklace in the pathogenesis of the hypersensitive reaction. In vitro ImmunoCap IgE assay was positive to latex (1.30 kUa/L), subsequent patch testing for contact dermatitis provoked a reaction for benzophenone‐4, (a UV stabalizer) and Fourier Transform Infra Red spectroscopy identified the elastic as ethylene propylene rubber, containing additional unidentified compounds. Allergy to natural rubber latex occurs in as many as 6% of Americans and Australians. Around three million American residents are thought to scuba dive each year. Recreational divers are, therefore, advised to check such necklaces, which are typically worn around the throat, for frayed ends and exposed rubber filaments.

## Introduction

Technical diving, where divers exceed commonly accepted recreational “no‐stop” depth‐time limits or enter overhead environments, has experienced explosive growth in this century. An essential element of technical diving is the principle of redundancy, including the requirement to carry two sources of breathing gas. A common method of carrying two regulators is to have the primary regulator in the mouth and the back‐up regulator attached to a necklace made of shock‐cord, surgical tubing or some other flexible material.

## The Case Report

A 49‐year‐old Australian male technical diver, now living in the USA, visited Nevada for a scuba diving trade show. On the day before the dive show, the diver and his dive buddy visited the Arizona side of Lake Mead and dived to 45 m depth, to photograph a narrow underwater canyon. The primary gas breathed was air and decompression was accelerated by switching to EANx50 during ascent, a breathing mixture containing 50% oxygen and 50% nitrogen. Maximum depth was reached 12 min into the dive and the total dive time, including decompression, was 39 min. The dive was made in side‐mount configuration in which two primary scuba tanks are distributed one on either side and the diver alternates breathing from either tank, to ensure approximately equal gas depletion. Accordingly, the diver wore a “double‐necklace” and to swap second‐stage regulators he would simply move both regulators on the single necklace from side to side. Whichever regulator was not in use thus sat against the diver's cheek, enabling rapid deployment should an emergency arise.

**Figure 1:**
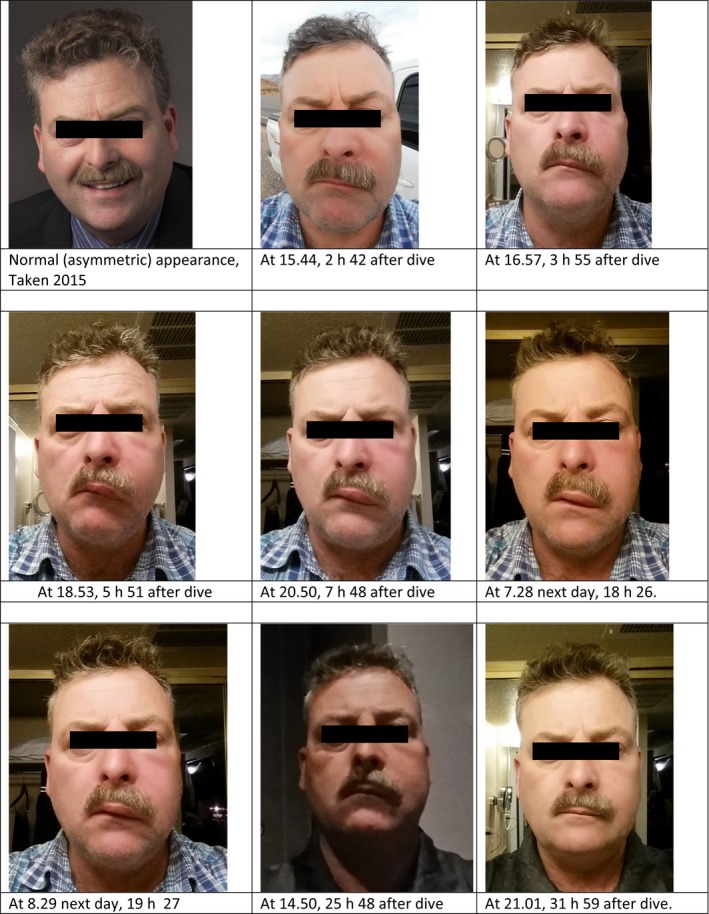
Progression of facial swelling.

At around 45‐min before diving the diver had consumed about 30g of 70% cocoa dark chocolate. Soon after surfacing, between 30 and 90 min, the diver drank two beers (approximately 5% alcohol by volume). At this time, the diver reported feeling a numb left upper lip but initially attributed this to the recent fit of a new silicone mouthpiece, coupled with nearly three‐quarters of an hour of diving in 20^°^C water. The pair repacked their dive gear into a car at around 120‐min post‐dive and departed for Las Vegas. By the time of departure the diver reported his left levator labii also feeling numb. During the drive, at 160 min after surfacing, the driver drew attention to visible swelling on the diver's left face. At that time the diver took a photograph (Fig. 1).

The diver reported no pain, merely mild numbness of the sort experienced after dental anesthetic. The swelling continued, by the evening extending from the left upper lip to the lower eye, and from the nose to the left cheek. Palpation indicated soft, fluid‐filled swelling. The diver ate dinner and reported neither difficulty maneuvering his tongue nor loss of taste.

The diver is a nonsmoker with a body mass index of 30. He reports regular exercise and has made approximately 2000 dives over the preceding 25 years without previous medical complication. Current medications are 10 mg of the ACE inhibitor lisinopril for hypertension, 20 mg of the statin atorvastatin for cholesterol reduction and 81 mg of aspirin per day for primary prevention against a cardiac event. He reports no history of adverse drug reactions, but does report mild symptoms of hay fever.

The diver conferred with medics from Divers Alert Network and initial discussions ruled out facial baroparesis, lymphatic decompression sickness, sialolithiasis, and barodontalgia. Although some sort of allergic reaction was suspected, the fact that it affected only one side of the face was initially mysterious. The swelling lasted 24 h before starting to abate and the diver reported returning to “almost normal” after 32 h.

**Figure 2:**
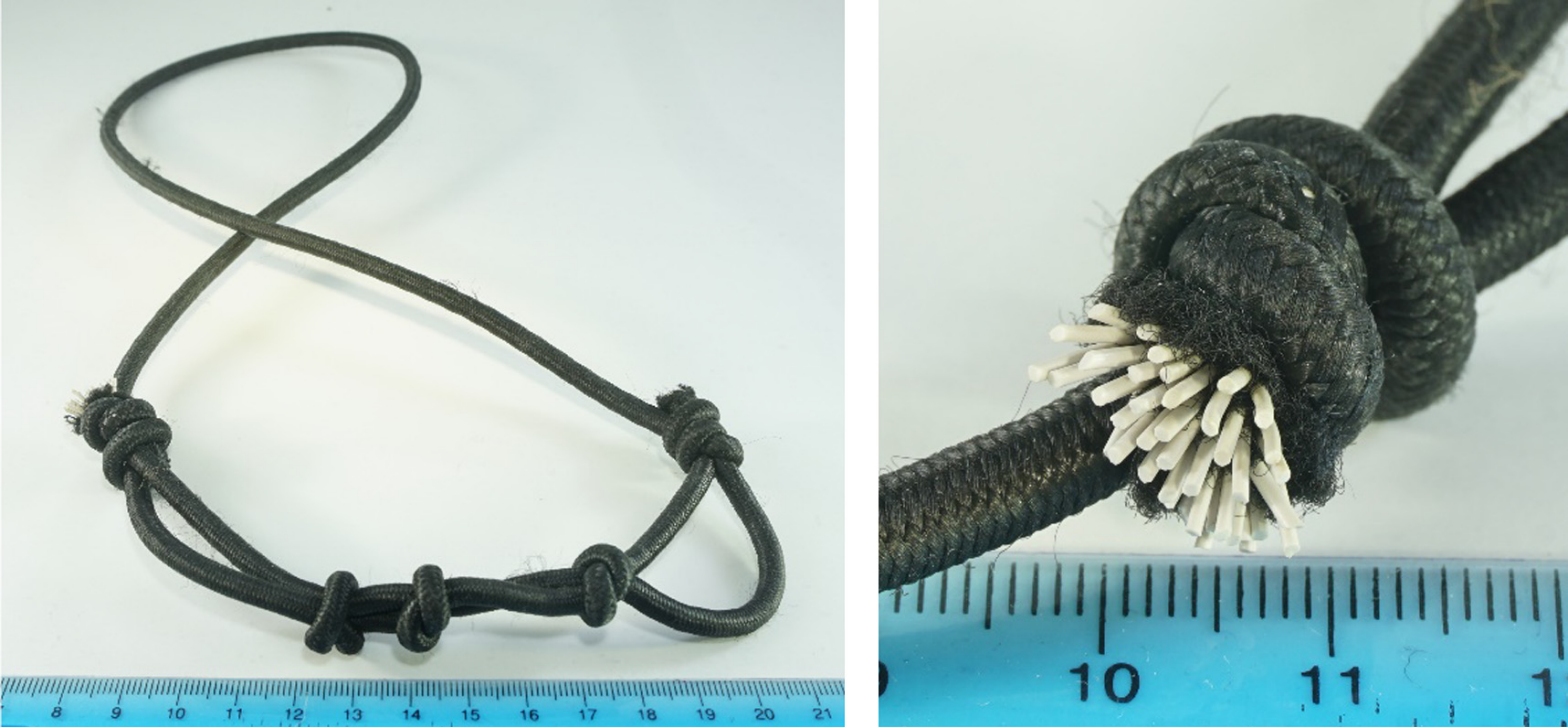
The necklace made of shock‐cord, with frayed end detail.

During the dive, the equipment that had come into contact with the diver's face included the drysuit neck‐seal, three scuba regulators with silicone mouthpieces, the necklace, mask, hood, oral inflator of the buoyancy control device, and two waterproof gloves. Although contact dermatitis to substances found in dive masks has been reported (Bergendorff and Hansson 2007), this seemed unlikely due to the relatively rapid time course and the unilateral symptoms. Both chocolate and beer were also assessed as potential factors, but through a process of elimination the home‐made necklace was identified as the likely culprit. Close inspection found the outer sheath worn at one end and the internal, white, rubber filaments extruding (Fig. 2). When the diver was at depth, these filaments would have been in contact with the diver's upper left lip while he was breathing from the left second‐stage regulator, and when the diver changed to the right‐hand second‐stage regulator the filaments would have been in contact with the mid‐left‐cheek.

One month after this incident the diver physically tested each piece of equipment against his face, (except the necklace), and then wore the same dive equipment (except the necklace) during a repeat 39‐min dive in freshwater in North Carolina, without provoking a similar reaction.

The rapid progression of symptoms suggests that this was not a Type IV immunologic contact dermatitis reaction. A blood test confirmed the diver has IgE‐mediated immediate hypersensitivity (Type (I) to latex (Latex IgE = 1.30 kUa/L, standard range < 0.35 kUa/L) (Phadia ImmunoCAP Total IgE, Phadia AB, Uppsala, Sweden) (Burkhart et al. 2015). A battery of 70 standard contact dermatitis test patches (allergEAZE, SmartPractice Canada) confirmed sensitivity to benzophenone‐4, (a UV stabalizer). Since the diver was found to be latex‐allergic, he was advised to limit exposure to latex products, particularly in healthcare scenarios. Fourier Transform Infra Red spectroscopy identified the elastic as ethylene propylene rubber, (a synthetic elastic), however, the filaments also tested positive for other unidentified compounds not found in the reference libraries checked.

## Discussion

This home‐made necklace made of shock‐cord is of the type commonly worn by technical divers, although necklaces for single regulators are by far a more common design. In this case, the diver's drysuit and bibbed hood kept the rubber filaments from being in contact with the diver's throat for an extended period. This particular necklace was constructed from 4 mm shock‐cord purchased from a franchise chain hardware store in 2012. It was subsequently used in around 100 dives, mostly in freshwater but occasionally in the sea. Over the 5 years since its initial deployment it had been subjected to environments ranging from near‐freezing water in lakes surrounded by snow, to balmy days at sea on dive boats, to sustained air temperatures of 40°C and high humidity in full sunlight. Some degradation of the rubber compound is, therefore, likely.

Approximately 12 million tons of natural rubber latex are produced annually. Immunologic and other types of adverse reactions to this ubiquitous material occur in as much as 6% of the general population of Australia and the US (Wu et al. 2016). IgE‐mediated immediate hypersensitivity (Type I) reactions can range in severity from localized edema to systemic anaphylaxis and death. Direct skin contact is the usual entry point for latex allergens, and sensitivity can increase with repeated exposure (Turner et al. 2012). It is possible that technical divers who routinely expose their throat or face to fraying rubber‐filled cord while scuba diving may be at increased risk of developing hypersensitivity to latex. Around 1%, or more than 3 million Americans are thought to engage in recreational diving each year (Sports and Fitness Industry Association, 2015). Therefore, technical divers employing home‐made necklaces constructed of rubber‐filled shock‐cord are encouraged to inspect them regularly and renew them when signs of degradation present. Those known to be latex‐allergic should use alternate materials for their necklaces.

## Conflict of Interest

Authors PB and JC are employed by Divers Alert Network, a not‐for‐profit company that sells scuba diving insurance and staffs a 24/7 diving emergency assistance telephone service. No other competing interests are declared. This study conforms with the CARE statement for case reports and signed informed consent was obtained from the diver.
